# Autotransplantation of a Strange Positioned Impacted Central Incisor in a surgically Prepared Socket: A Miracle Esthetic Concept

**DOI:** 10.5005/jp-journals-10005-1375

**Published:** 2016-09-27

**Authors:** Chandresh Jaiswara, Vinay K Srivastava, Neeraj Dhiman

**Affiliations:** 1 Assistant Professor, Department of Oral Surgery, Faculty of Dental Sciences Institute of Medical Sciences, Banaras Hindu University Varanasi, Uttar Pradesh, India; 2 Associate Professor, Department of Dentistry, Faculty of Dental Sciences, Institute of Medical Sciences, Banaras Hindu University, Varanasi Uttar Pradesh, India; 3 Assistant Professor, Department of Oral Surgery, Faculty of Dental Sciences Institute of Medical Sciences, Banaras Hindu University Varanasi, Uttar Pradesh, India

**Keywords:** Ankylosis, Autotransplantation, Fresh prepared bony socket.

## Abstract

Esthetics is a prime concern for a young lady. Any anomaly in the anterior tooth may create anxiety and depression. This anxiety and depression may hamper her married life and overall personality. This case report reveals an unerupted right central incisor situated in a strange position, creating space in the maxillary anterior region and giving an unesthetic appearance. Autotransplantation is a method of choice for a strangely positioned impacted central incisor in a new appropriate site. This method offers a new treatment option for some clinical situations if orthodontic approach is not possible. It permits tooth movement to a distant or the opposite side of the same dental arch as well as to the opposite jaw. This procedure also offers potential benefits of reestablishment of normal alveolar process development, esthetics, functions, and arch integrity. This procedure has the potential to become a viable alternative treatment plan for young patients of low socioeconomic status, allowing the reestablish-ment and restoration of a missing tooth and their functions. This article discusses methods of auto-reimplantation of a tooth in a fresh surgically prepared socket, its biological principle, and establishment of functions, esthetics, and phonetics.

**How to cite this article:**

Jaiswara C, Srivastava VK, Dhiman N. Autotransplantation of a Strange Positioned Impacted Central Incisor in a surgically Prepared Socket: A Miracle Esthetic Concept. Int J Clin Pediatr Dent 2016;9(3):269-272.

## INTRODUCTION

Autotransplantation pertains to the repositioning of autogenous erupted, semi-erupted, or unerupted tooth from one site to another in the same individual.^1^ Transplantation site may be either an extraction socket or a new surgically prepared alveolus to receive a tooth.^2,3^ Transplantation plays a key role in the restoration of functions of a missing tooth. Autogenic transplantation was first described by Swedish dental surgeon Vidman in 1915.^4^ Transplantation offers a new horizon for bone induction and reestablishment of a normal alveolar process in addition to tooth replacement and restoration of esthetics and functions, arch integrity, and phonetics. If the transplant fails, there will be an intact site to receive an implant. A prerequisite for this method is a thorough knowledge of factors influencing the success rate (biological principle).

## CASE REPORT

**Fig. 1:**
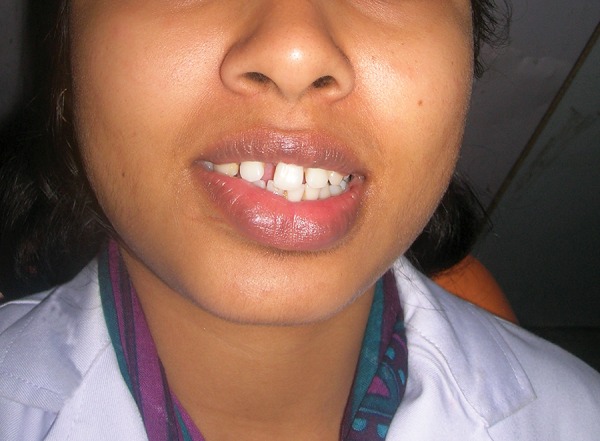
Spacing in anterior teeth and unesthetic smile appearance

**Figs 2A and B:**
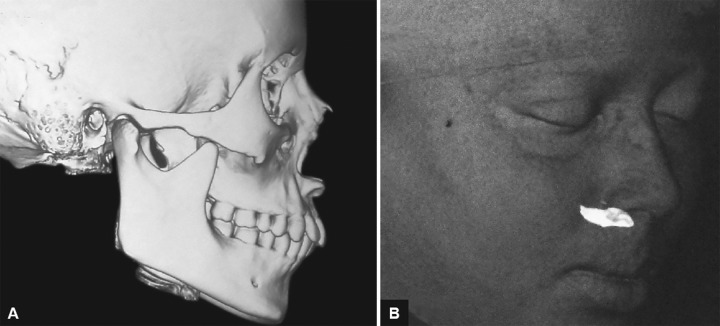
Computed tomography scan shows strange position of maxillary right central incisor and soft tissue recreation on CT scan and strange tooth position

A 19-year-old female presented to the dental OPD IMS, BHU, with complaint of nonerupting right central incisor. She had no significant medical history. Intraoral examination revealed a normal tooth with a narrow 3.5 mm space for right central incisor due to an unerupted right central incisor (Fig. 1). On radiographic evaluation, an impacted central incisor was noted. To know the exact position of the impacted tooth, a computed tomography (CT) scan was advised. Computed tomography scan and reconstruction of soft tissues on CT scan showed a strange positioning of the impacted tooth (Figs 2A and B).

**Fig. 3:**
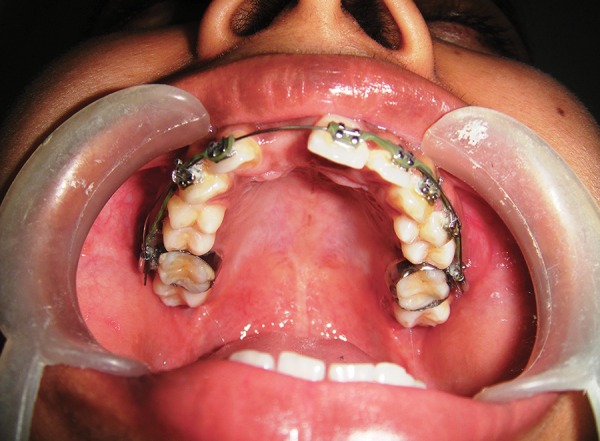
Adequate anterior space was created by fixed appliance

**Fig. 4:**
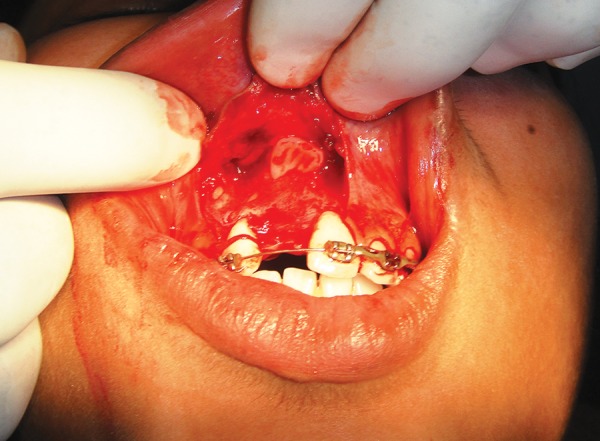
Tooth crown was surgically exposed

Orthodontists were consulted and they had denied repositioning of the central incisor in its appropriate space by means of orthodontic movement probably due to the curved root and the possibility of damage to the nasal floor and antrum.

### Treatment Plan

The right central incisor space was recreated by means of orthodontic movement (Fig. 3). After acquiring adequate space for the right central incisor, we planned for surgical repositioning or autotransplantation of the impacted central incisor into a fresh surgically prepared socket. Blood investigation reports were within normal limits.

After surgical part preparation with betadine solution, an incision was made from the distal surface of right lateral incisor to the distal surface of left maxillary central incisor. The flap was raised with the help of a periosteal elevator by considering the normal anatomical structure. The raised flap exposed the crown of the impacted central incisor (Fig. 4), which was positioned in an anteriolateral direction with the root tip near the nasal antrum as noted in the CT scan.

**Fig. 5:**
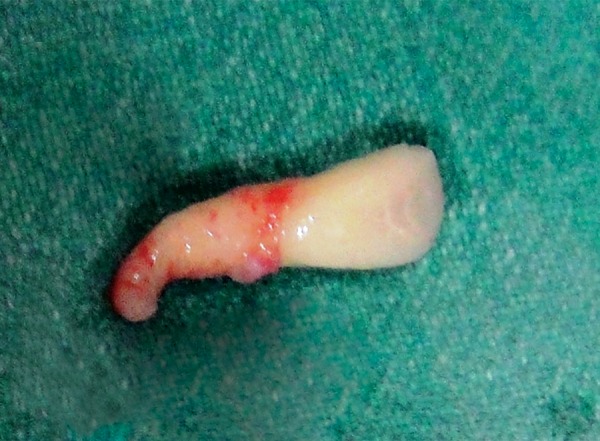
Extracted maxillary central incisor with curved root tip

A small groove was made around the neck of the impacted central incisor with the help of a small bone-cutting rotary bur with adequate cooling for a better forceps grip. A gradual small rotary force was applied on the neck of the impacted tooth to prevent fracture of the tooth neck or the root. This technique also helps us to expand the alveolar bone, and the tooth was removed (Fig. 5) and placed in sterile normal saline.

### Socket Preparation (Fig. 6)

The tooth socket was prepared with the help of a bone bur with adequate cooling so that bone physiology was not hampered. This step also helps in postoperative healing, since the bony socket was freshly prepared by splitting of the buccal cortical plate along with slight elevation of the cortical plate and a bone bur was used to make a socket in the cancellous bone. Viable periodontal ligaments are required for tooth-bone attachment otherwise the tooth ankyloses with bone and physiological root resorption starts. Diameter of the tooth root was measured and an adequate root angulation was marked.

**Fig. 6:**
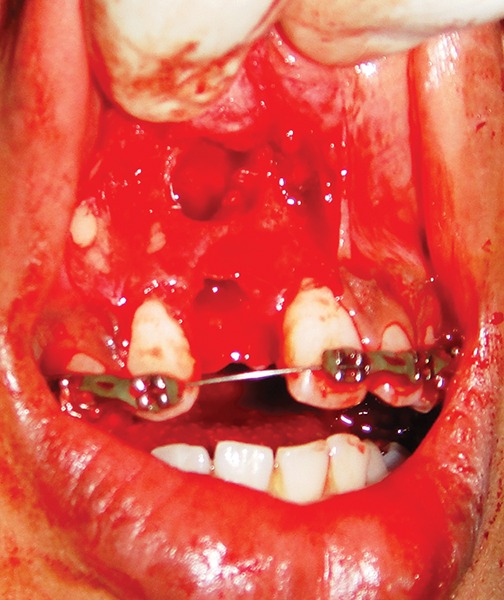
Surgically prepared socket

### Tooth Preparation

- *Root surface preparation:* Root was fully formed. Since we promote attachment, viable periodontal ligament is significant in the success of autotransplantation.
- *Endodontic treatment:* Since the root apex was fully formed and curved, we decided to do RCT and cut the curved part of the apex for a smooth socket insertion path and orthograde obturation was done extraorally and the apex was sealed with GIC cement.

**Fig. 7:**
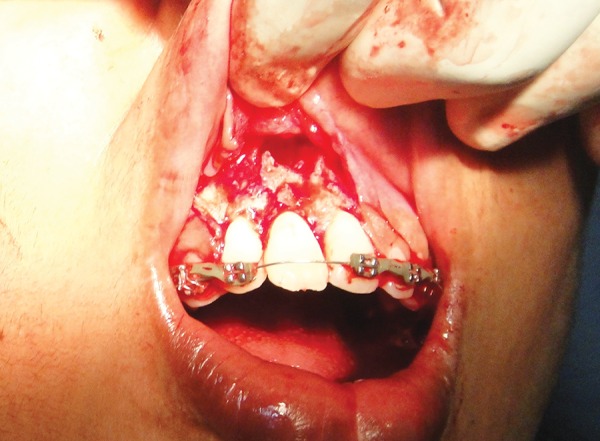
Endodontically treated tooth positioned in surgically prepared socket and splinted with semi-fixed splint

Placement of the extracted impacted tooth into a fresh prepared bony socket (Fig. 7): The extracted tooth was transplanted into the prepared socket gently within 15 minutes after extraction. Precaution was taken to stimulate bleeding into the socket to receive the transplanted tooth and disocclude the transplanted tooth from occlusion to promote healing.

**Fig. 8:**
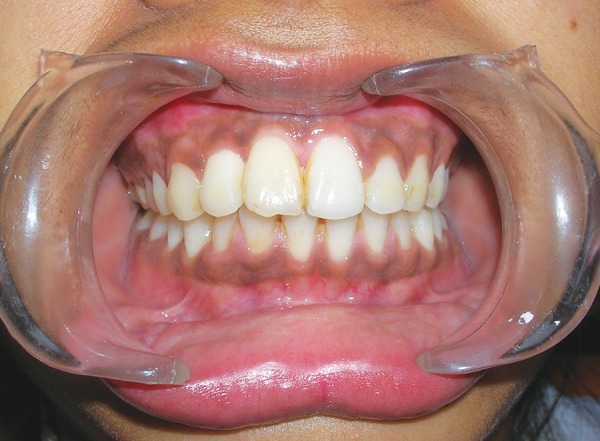
After 3 months of surgery, esthetics is reasonably improved. See the gum line

*Semi-fixed Splint:* A splint was made to fix the replanted tooth for 4 weeks and the raised flap was repositioned with 3.0 silk sutures. After 4 weeks, the splint was removed and the tooth was polished (Fig. 8) to remove composites (the splinting material).

## EVALUATION BY RADIOGRAPH AND CLINICAL SYMPTOM

**Fig. 9:**
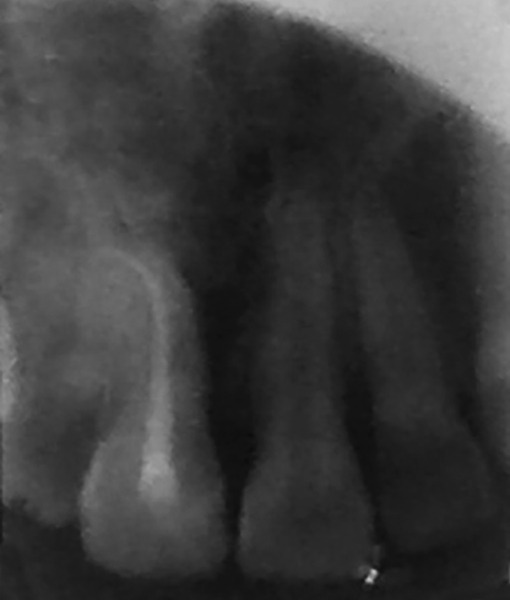
IOPA radiograph showing slight resorption on mesial surface

**Fig. 10:**
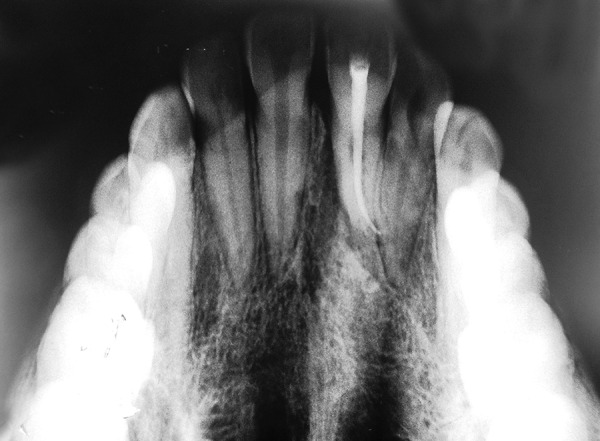
One-year postoperative occlusal radiograph is showing slight resorption on apex of tooth. It might damage the cemental surface at the time of extraction

**Fig. 11:**
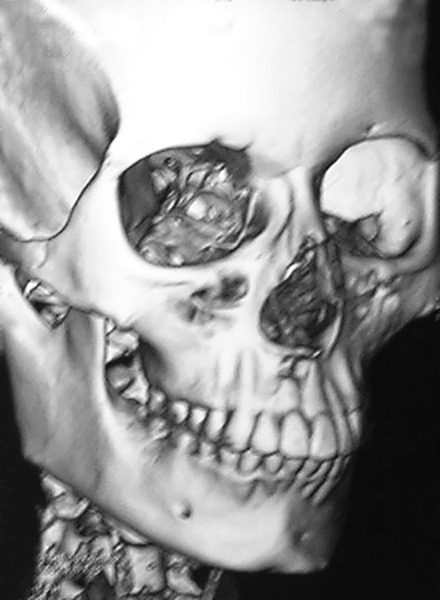
Compare the pre- and postsurgery CT. See the difference between locations of strangely positioned area

Slight resorption areas were detected on the mesial surface of transplanted tooth root on the intraoral periapical radiograph (Fig. 9). After 1 year, in postoperative occlusal view (Fig. 10), slight resorption was seen on mesial surface in cervical region, and periodontal space seems to be obliterated below the resorption area. On distal surface, normal periodontal space was seen. The tooth was asymptomatic after 1 year of autotransplantation. Postoperative occlusal view also showed slight apex root resorption; it might be due to damage to the curved root cemental surface at the time of extraction. Rest of the root surface anatomy seems to be normal. Postoperative CT scan (Fig. 11) of the anterior region showed normal alveolar elevation and healing area of the extracted impacted central incisor. Normal gingiva and normal gum line developed in the region of autotrans-plantation (Fig. 8).

## DISCUSSION

Esthetics is a prime concern for a young woman. Any anomaly in the anterior tooth may create anxiety and depression. This anxiety and depression may hamper her married life and overall personality. In the above case, the woman was an unmarried postgraduate and much worried about her personality, looks, and upcoming married life, and the behavior of her colleagues toward her. The type of healing of a transplanted tooth is dependent on the surface area of the damaged root to be repopulated. If the damaged PDL is small, the healing would be achieved by cemental healing. If the damaged PDL surface is large, some of the root surface will be resorbed, followed by apposition of bone (Ankylosis). Thus, root resorption will develop. The contributions of progenitor PDL cells on the recipient’s fresh extraction sockets also account for the higher success rate for fresh extracted recipient sockets as compared to artificially drilled ones.^5^

## CONCLUSION

Autotransplantation of strangely positioned impacted central incisor on a new appropriate site offers a new treatment option for some clinical situations. It permits tooth movement to distant or opposite sides of the same dental arch as well as to the opposite jaw. This procedure also offers potential benefits of reestablishment of normal alveolar process development, esthetics, functions, and arch integrity. This procedure has a potential to become a viable alternative treatment plan for a young patient of low socioeconomic status, allowing the reestablishment of restoration of missing teeth and its functions.

Precautions should be taken during extraction and damage to the cemental surface should be minimum. This procedure enhances success of auto transplantation in a surgically prepared socket.
